# To Live or Die: What to Wish at 100 Years and Older

**DOI:** 10.3389/fpsyg.2021.726621

**Published:** 2021-09-10

**Authors:** Lia Araújo, Laetitia Teixeira, Rosa Marina Afonso, Oscar Ribeiro

**Affiliations:** ^1^Center for Health Technology and Services Research (CINTESIS), Porto-Aveiro, Portugal; ^2^Department Psychology and Educational Sciences, School of Education, Polytechnic Institute of Viseu, Viseu, Portugal; ^3^Department of Population Studies, Institute of Biomedical Sciences Abel Salazar, University of Porto, Porto, Portugal; ^4^Department Psychology and Education, University of Beira Interior, Covilhã, Portugal; ^5^Department of Education and Psychology, University of Aveiro, Aveiro, Portugal

**Keywords:** will to live, centenarians, valuation of life, religion, PT100, longevity

## Abstract

Previous research has shown that will to live is a strong predictor for survival among older people, irrespective of age, gender, and comorbidities. However, research on whether life at age 100 is perceived as worth living is limited. The available literature has presented evidence for good levels of positive attitudes and life satisfaction at such an advanced age, but it has also suggested that a longing for death is common. This study aimed to add to the existing data on this matter by exploring centenarians' will to live and the associated factors. The sample comprised 121 centenarians (mean age, 101 years; SD, 1.63 years), 19 (15.7%) of whom were males, from two centenarian studies (PT100). Answers to open questions were analyzed to identify the centenarians' will to live and the reasons behind it. Three groups were created (willing to live longer, not willing to live longer, no clear positioning) and further analyzed in terms of sociodemographic characteristics, health status, social functioning, and well-being. Of the total sample, 31.4% expressed willingness to live longer, 30.6% did not, and 38% presented no clear positioning. The presence of the Catholic religion (God) was referred for centenarians in all three groups. Annoyance, uselessness, loss of meaning, disconnection, and loneliness were the most common justifications for being reluctant to live longer. Positive valuation of life and good self-rated health, followed by having a confidant and reduced pain frequency, were the factors associated with being willing to live longer. The results of the study contribute to the understanding of the psychological functioning of individuals with exceptional longevity, particularly concerning the factors behind willingness to live at such an advanced age.

## Introduction

Due to the phenomenon of population aging, the length of human life has become of interest in aging and gerontological research. Particularly in developed countries, reaching 100 years of age is becoming more common (Teixeira et al., 2020), and some individuals, known as supercentenarians, will even live beyond 110 years old. This increase in life expectancy and longevity is the result of significant advances in medical, social, political, economic, and cultural domains that together have improved health, nutrition, and sanitation conditions (Mathers et al., 2015). However, this delay of mortality raises important questions about quality of life in the later years of those who achieve exceptional longevity. Although the general consensus is that living longer will only be desirable if lived with meaning, purpose, and good quality of life, the clinical, bioethical, and social implications of extended life expectancy have raised various debates (e.g., Serra et al., 2011).

Research on the desire to reach advanced ages has shown similar results across different age groups and countries. For instance, in a study of 715 university students in Austria, Norway, Poland, and Russia, Bowen et al. (2020) found that 25.8% of the participants wanted to live 100 or more years, while the great majority (74.2%) did not. Of the participants, 21.2% wanted to live fewer than 80 years, and 53% wanted to live to their 80s or 90s. In another study with a larger sample (1631) of young and middle-aged adults in the United States, Bowen and Skirbekk (2017) found that 26.4% preferred a life expectancy of 100 or more years old, 24.6% preferred a life expectancy in the 90s, and 31.9% preferred a life expectancy in the 80s.

Age (being older) has been shown to affect people's attitudes toward living an extremely long life (Huohvanainen et al., 2012; Karppinen et al., 2016). When asked about their desire to reach 100 years old, 32.9% (aged 75–96; Karppinen et al., 2016) and 37.2% (aged 72–88; Huohvanainen et al., 2012) of community-dwelling older adults from Finland wanted to live to be 100. Karppinen et al. (2016) also found that gender (male) and subjective health (positive) significantly impacted the desire to live longer. Furthermore, a qualitative analysis of the reasons why the oldest-old participants wanted to live to 100 showed the importance of health and functioning, as many of the participants wished to live longer if they could remain independent—that is, “as long as their health remains good” (Karppinen et al., 2016, p. 547). The participants who wanted to live to 100 also displayed positive attitudes (e.g., curiosity, love of life, belief) and rational reasons for living longer (e.g., significant roles, offspring).

Interestingly, when studying a sample of 115 centenarians' close family members (aged 25–86 years; mean, 64.97), Brandão et al. (2019) found that 56.5% wanted to reach 100. Thus, having close contact with a centenarian may have a positive influence on people's attitudes toward living longer. Still, the family members stated that conditional circumstances related to health and caregiving were required to make living to 100 desirable. These included, among others, having good physical health, not being bedridden, being capable of maintaining independence performing activities of daily life (ADL), having the ability to express their wishes (good cognitive status), and having family support and/or someone who provided care in case of dependency, which would allow them to continue living at home. Indeed, research on children's perceptions of challenges relating to the end of life of their centenarian parents showed that it seems to be a potential double confrontation for the offspring who are often old adults themselves, i.e., “some adult children reported that observing the centenarian was like looking into a mirror and a kind of preview of their own possible challenges” (Eggert et al., 2020, p. 7).

In a review of available theoretical and empirical work about motivation for longevity across the life span, Lang and Rupprecht (2019), identified that people have different profiles, which can be divided in three mindsets: (i) an essentialist, based on an infinite life, (ii) a medicalist, appraising aging as being primarily based on quality of health, and (iii) a stoicist mindset that associates longevity and lifetime extension with the experience of grace and meaning. The authors argued that longevity motivation depends of determinants related to context, health functioning, and personal belief systems, that there could be change in longevity motivation over time and that the mindsets have differential behavioral consequences in what ways individuals want to approach old age.

Despite the available literature on the desire to become a centenarian in several populations (e.g., younger adults, centenarians' family members) and longevity extension, whether centenarians themselves still want to live longer remains undetermined, especially since despair, depressive symptoms, and large suicide rates have been documented in this population (Shah et al., 2014). Will to live is an important indicator of well-being that can be perceived as a basic need, a goal, and a drive (Carmel, 2012). Will to live reflects a focus on the present and a person's existential motivation to live (Bornet et al., 2021). Lawton et al. (1999) presented will to live as the most concretely anchored cognitive outcome of a dynamic cognitive-affective thought process in which people weigh a variety of inputs that affect their psychological well-being. It is influenced by external and internal factors and is expressed physiologically, psychologically, and socially (Lawton et al., 1999; Carmel, 2012). Furthermore, will to live has a great association with psychological variables like resilience and life satisfaction (Bornet et al., 2021). Lawton et al. (1999) proposed that years of desired life are mediated by an intervening cognitive-affective schema, which they designated as “valuation of life” (VOL). This schema includes hope, futurity, purpose, meaningfulness, persistence, and self-efficacy as its main core constructs (Lawton et al., 2001). In his well-known Holocaust memoir, Man's Search for Meaning, Frankl (1963) associated will to live with purpose; using Nietzsche's words, he stated that “he who has a why to live for can bear with almost any how” (p. 84).

Although heterogeneity is expected in the functionality of those of advanced age, most centenarians face high constraints in terms of physical, sensory, and cognitive function (Serra et al., 2011). However, many centenarians are still able to maintain good levels of life satisfaction, positive affect, and happiness (Cheng et al., 2021). Psychological strengths, such as optimistic outlook (Jopp and Rott, 2006), positive life attitudes (Wong et al., 2014; Kato et al., 2015; Mackowicz and Wnek-Gozdek, 2016), purpose (Araújo et al., 2016), and existential beliefs (Araújo et al., 2017), may be of particular importance for centenarians' well-being. Additionally, faith and social relationships seem to affect the way centenarians experience and perceive their old age, regardless of personal limitations and differences (Mackowicz and Wnek-Gozdek, 2016). In a study investigating the relationship between meaning in life, will to live, and age-associated health restrictions (e.g., number of diseases), Jopp et al. (2017) found that both meaning in life and will to live have strong, direct effects on well-being. Health factors were, in comparison, less important or non-significant.

Understanding older adults' will to live is important since they are increasingly aware of the approaching end of life and face cumulative losses in almost all domains of life (Carmel, 2012). Nevertheless, will to live has not been a main focus of research; a greater number of studies have investigated attitudes toward death and dying, especially in end of life care. Empirical research of the oldest-old (Fleming et al., 2016) and older adults without a serious medical condition (Van Wijngaarden et al., 2015) is limited. A recent study of German and Portuguese centenarians reported that a notable portion of the participants did not think about the end of their lives; however, most of these centenarians experienced health or social problems (Boerner et al., 2019). This study used both quantitative and qualitative approaches to deepen the available scientific understanding of will to live in extreme longevity and to explore its associated factors.

## Methods

### Data Collection

The data for this study came from two centenarian studies - the Oporto Centenarian Study (PT100) and the Beira Interior Centenarian Study (PT100 Beira Interior)—which were conducted in two distinct geographical regions of Portugal, each one with an area of ~60 km^2^. A total of 291 individuals aged 100 years and older between December 2013 and December 2014 were identified through voter registration files, churches, nursing homes, local media newspapers, and snowball sampling. Of the 291 centenarians, 50 were excluded because they died in the interim or because their relatives refused their participation due to advanced dementia, other major health problems, or lack of interest in the study. The 241 participants were interviewed face-to-face, and a sample of 121 centenarians who were not affected by severe cognitive impairment and who answered the questions about their thoughts and own perceptions required for the study were included.

The data were collected during one or two sequential interview sessions either with the centenarian and/or a proxy respondent. Following recommended strategies (Sachdev et al., 2012), age validation was performed by a protocol entailing personal identity document verification (e.g., birth certificate) and milestones assessments (e.g., wedding date, date of firstborn, subsequent birthdates of children). An informed consent previously approved by the National Commission on Data Protection was used. More information about the methodological procedures of both centenarian studies can be found in Ribeiro et al. (2015).

### Measures

#### Socio-Demographic Characteristics

The following variables were considered in this set: age, sex (male/female), education (having/not having formal education), and living in an institution (yes/no).

#### Health Condition

The following objective and subjective health measures were determined: pain frequency (never, seldom, sometimes, often/always), presence of physical fatigue (yes/no), and self-rated health (SRH). To determine SRH, the following question was asked: “In general, would you say your health is…?” Five response options were given: excellent, very good, good, reasonable, and bad. The responses were scored 1 for a bad SRH, 2 for a reasonable SRH, and 3 for a positive SRH (i.e., excellent, very good, or good).

The total number of self-reported health conditions was obtained from a list of age-related problems commonly observed in the older population, including high blood pressure, a heart condition, diabetes, chronic lung disease, ulcers or other serious stomach issues, cirrhosis or other liver problems, a kidney condition, frequent urinary infections, incontinence, prostate problems, problems with vision or hearing, arthritis, osteoporosis, stroke, cancer, pneumonia, and falls, among others. Functional capacity was assessed through the Older Americans Resources and Services (OARS) Multidimensional Functional Assessment Questionnaires (Fillenbaum and Smyer, 1981). This questionnaire includes seven items that assess basic ADL (e.g., the capacity for walking, bathing, eating, going to the toilet) and seven items to assess instrumental activities of daily living (IADL; e.g., the capacity to use the telephone, go shopping, do housework, prepare meals). The participants were asked to rate how much difficulty they had performing each of these activities on a three-point scale (2 = no difficulty; 1 = can complete the activity with some help; 0 = cannot complete the activity without help). The seven items of ADL and the seven items of IADL were summed to obtain the total scores of each domain, with a higher score indicating greater independence.

#### Social Functioning

The following questions from the Social Resources subscale of the OARS (Fillenbaum and Smyer, 1981) were considered: the number of visits the centenarians had on a regular basis (none; 1–2; 3–4; 5 or more), how often the centenarian felt lonely (many times, sometimes, almost never, or never), and the time spent with people the centenarian did not live with (none, once/week, 2–6 times/week, once/day, or more). Four questions from the Lubben Social Network Scale (Lubben, 1988) were also used: the number of relatives and friends that the centenarian saw or heard from at least once a month and the number of relatives and friends that the centenarian has as a confidant, i.e., that could talk to about private matters (none; 1–2; 3+).

#### Well-Being

The Satisfaction with Life Scale (SWLS; Diener et al., 1985) is a short, five-item instrument that was designed to measure global cognitive judgments of satisfaction with one's life. The format of the questionnaire was modified after reported methodological constraints in assessing centenarians who described their difficulties in understanding self-referent statements and using five or six answering categories (Jopp and Rott, 2006). To avoid these difficulties, the questionnaire was changed from statements to questions, with a response of 0 being no, 1 being in between, and 2 being yes.

The Positive Valuation of Life Scale (Positive VOL; Lawton et al., 2001) is a 13-item scale that was formulated to examine the factors that may influence a person's will to continue to live and affect end-of-life attitudes and behaviors. In the Portuguese version used in this study, two factors were identified: existential beliefs and perceived control (Araújo et al., 2015). The answering format was changed from five to three options (0 = no, 1 = in between, and 2 = yes) due specific difficulties in assessing centenarians (see Jopp and Rott, 2006).

#### Will to Live

The outcome variable was derived from the qualitative analysis of responses to two open questions (To what age would you like to live? and Do you long for death?). The responses were coded as yes (willing to live longer), no (not willing to live longer), and no indicators of will (without a clear positioning).

### Analysis

The answers given to the questions were audiotaped, transcribed, and subjected to qualitative analysis. The first and last authors carefully read all the transcripts and defined the three major categories of the centenarians' will to live: willing to live longer, unwilling to live longer, and without a clear positioning. Then, the two authors met to match their independent ratings and discuss the differences and interpretations of the data.

In the next phase, the transcripts were examined to identify common themes using open coding, clustering, and theme identification (Hsieh and Shannon, 2005). These themes regarded the common reasons presented for willingness to live longer (or not), as well as the reasons for not presenting a clear positioning. Then, both authors independently analyzed the data and discussed discrepancies until reaching consensus. All answers were carefully reread, and final categorization of the emergent themes was defined (Braun and Clarke, 2006). Final categories were later discussed with the other authors.

Quantitative and descriptive analyses were used to characterize the sample according to sociodemographic characteristics, health status, social functioning, and well-being. To calculate the total score of the scales (e.g., satisfaction with life), the items missing data were replaced, and the mean was considered when at least 50% of the items were completed. The comparison of groups, which was defined by the outcome “will to live,” was performed using a Chi-square test or Fisher's exact test and one-way ANOVA. In all quantitative analyses, a significance level of 0.05 was considered. All analyses were performed in IBM SPSS software (version 26.0).

## Results

### Sample Characteristics

The sample comprised 121 centenarians with an overall mean age of 101 years (*SD* = 1.5 years); 19 (15.7%) were male. A majority of the centenarians (45.5%) had no formal education, and 40.5% were institutionalized. Regarding health status, 44.6% reported that their health was perceived as being “good, very good, or excellent,” while 42.6% reported physical fatigue, and 17.5% reported never having pain. The mean scores of ADL and IAL were 3.71 (*SD* = 3.21) and 8.23 (*SD* = 4.26), respectively. Finally, the average number of diagnoses was 3.26 (*SD* = 1.94). Concerning social function, 36.2% of the centenarians had 5 or more visits, 51.0% reported missing people around them, and 25.7% never felt alone. The mean score of the SWLS was 6.54 (*SD* = 1.93). The mean scores of the Positive VOL Scale, factor 1 (existential beliefs), and factor 2 (perceived control) were 16.7 (*SD* = 6.0), 9.39 (*SD* = 3.58), and 7.32 (*SD* = 2.81), respectively.

### Overview of Qualitative Responses

Of the sample, 31.4% expressed their willingness to live longer (group 1), 30.6% did not (group 2), and 38% presented no clear positioning (group 3). Explanations for being (un)willing to live were given by 63% (*n* = 38) of those in the first group and by 78% (*n* = 37) of those in the second group; the other participants did not give any justification for their answers. From the third group (*n* = 46), 38 participants referred to aspects related with God; this were the only topic of reference for their answers.

The reference to God appeared in all three groups but with different meanings. Those who reported wanting to live longer asked God to provide them additional time (“Living another month or two is already good, but I put it in God's hands”). Those reporting being unwilling to live longer asked God to take them/asked God for death (“I would like to die, but God gave me this punishment of living so many years, and I must accept it”). Finally, those who did not demonstrate a clear presence/absence of will to live stated that their future was in God's hands (“We do not command anything, only God knows”). Tables 1, 2 present the reasons given by groups 1 and 2, respectively, as well as the number of quotations in each category and illustrative quotations.

**Table 1:** Reasons for willingness to live longer and the number of quotations for each.

**Category (*n*)** ^**a**^	**Examples of quotations**
God (14)	I'm ready as soon as God understands [it's the time], but I want to live at least until I am 103 years old.
Family (10)	I would like to see my grandson's [university] graduation.
Conditional wish: if in the presence of similar functioning levels and without being a burden (3)	[Would like to live] one more year […] but without being a burden to others or being bedridden.
Enjoying living (3)	Now that the good weather comes [springtime approaching], I want to live!

a *Frequencies were not mutually exclusive*.

**Table 2:** Reasons for being unwilling to live longer and the number of quotations for each.

**Category (*n*)** ^**a**^	**Examples of quotations**
God (10)	I would like to die, but God gave me this punishment for living, and I have to accept it.
Annoyance (9)	Time passes so slowly, especially the nights; it seems that the day never comes again.
Uselessness (6)	(Because) a person wanting to work and not being able to […] is not worthy.
Loss of meaning (5)	I often ask myself “What am I still doing here?”
(Social) disconnection and loneliness (5)	I go to the street to have a coffee […] people always come, look at me but I don't know anyone.
Sense of burden (3)	I cannot do anything; I am here just to overwhelm [the others].
Dependency (3)	Because I am getting older, with less strength, and I'm no longer able to drag myself.
(Fear of) suffering (3)	I wanted to die so I do not have to be here in suffering. I have a lot of pain.
Living outside my own home (3)	I am very scorned here. I didn't need to be here at the nursing home.
Loss of family members (1)	I wish I had already died when my little children died.

a *Frequencies were not mutually exclusive*.

In the first group (i.e., those willing to live longer), the most frequent reasons besides those related to God concerned family (*n* = 14), such as the wish to meet new great-grandchildren or see a grandchild's achievements (e.g., wedding, university graduation). Further reasons were conditionally related to the centenarian's future functioning in the sense that the participants only wanted to live longer if they were in the same condition they were at the time of the interview, without being a burden to others. Three participants reported enjoying life, in the sense that they want to live longer to appreciate the good things of life.

The centenarians in group 2 (i.e., those unwilling to live longer) presented a greater diversity of reasons for their answers. Along with aspects related to God, important reasons included annoyance (*n* = 14), uselessness (*n* = 6), and loss of meaning (*n* = 5). The participants stated that they did not desire to live longer because “time passes so slowly” and because every day was alike. This reason was related to a feeling of being useless; several participants complained about doing nothing/not being able to work or about the loss of meaning due to lack of purpose in life. Disconnection and loneliness constituted other reasons; some participants felt detached from the place they lived (“I go to the street […] I don't know anyone anymore”) or lonely, especially those who spent long periods of time alone. Dependency, sense of burden, and suffering (or the fear of being in suffering) were referenced each as reasons by three participants and related to situations of lacking functionality and pain. Three participants (who lived in nursing homes) stated that living outside their home was the reason they were unwilling to live longer. Lastly, one participant mentioned the loss of his children as the life event that made him lose all desire to live longer (Table 2).

### Factors Associated With Will to Live

Table 3 presents the relationships between the three groups and sociodemographic characteristics, health status, social functioning, and well-being. Sociodemographic characteristics were not associated with will to live. Regarding health status, pain frequency and SRH were significantly associated with will to live. Group 2 (unwilling to live longer) presented higher pain frequency (50.0% as often/always) compared to the other groups (22.9% in group 1 and 27.5% in group 3). Additionally, 58.3% of the individuals who were willing to live longer expressed having a good, very good, or excellent SRH, while only 27.3% of individuals unwilling to live longer presented such a positive SRH. Regarding social functioning, having a friend confidant was the only variable associated with will to live. In the group unwilling to live longer, a higher percentage (87.5%) did not have any confidant compared to the other two groups (42.9% in the group willing to live and 52.6% in the group with no clear positioning). For well-being, positive VOL was significantly associated with the outcome variable. Group 1 presented higher mean scores of positive VOL (mean = 20), including factor 1 (mean = 11.1) and factor 2 (mean = 8.84), compared to the other two groups.

**Table 3:** Distribution of sample characteristics by total and will to live groups.

	***n***	**Total**	**Will to Live**			***p***
		***n* (%) or mean (SD)**	**No** ***n* (%) or mean (SD)**	**Yes** ***n* (%) or mean (SD)**	**No clear positioning** ***n* (%) or mean (SD)**	
Total	121	121 (100.0)	37 (30.6)	38 (31.4)	46 (38.0)	-
**Sociodemographic characteristics**						
Age, mean (SD)	121	101.0 (1.5)	100.9 (1.5)	101.0 (1.4)	101.2 (1.5)	0.665^**^
Sex [male]	121	19 (15.7)	4 (10.8)	8 (21.1)	7 (15.2)	0.473^*^
Education [no formal education]	121	55 (45.5)	15 (40.5)	17 (44.7)	23 (50.0)	0.687^*^
Institutionalized [yes]	121	49 (40.5)	17 (45.9)	15 (39.5)	17 (37.0)	0.701^*^
**Health condition**						
Pain frequency	109					0.040^*^
Never		19 (17.4)	4 (11.8)	10 (28.6)	5 (12.5)	
Seldom		22 (20.2)	4 (11.8)	10 (28.6)	8 (20.0)	
Sometimes		32 (29.4)	9 (26.5)	7 (20.0)	16 (40.0)	
Often/always		36 (33.0)	17 (50.0)	8 (22.9)	11 (27.5)	
SRH	112					<0.001^*^
Bad		20 (17.9)	14 (42.4)	3 (8.3)	3 (7.0)	
Reasonable		42 (37.5)	10 (30.3)	12 (33.3)	20 (46.5)	
Good, very good, excellent		50 (44.6)	9 (27.3)	21 (58.3)	20 (46.5)	
IADL, mean (SD)	118	3.71 (3.21)	2.99 (2.56)	3.86 (3.49)	4.18 (3.39)	0.240^**^
ADL, mean (SD)	121	8.23 (4.26)	7.63 (4.16)	8.04 (4.53)	8.87 (4.13)	0.402^**^
Number of health conditions, mean (SD)	121	3.61 (1.94)	3.30 (1.61)	4.00 (2.19)	3.54 (1.94)	0.281^**^
Physical fatigue [yes]	115	49 (42.6)	17 (51.5)	15 (39.5)	17 (38.6)	0.471^*^
**Social functioning**						
Number of visits	105					0.457^***^
None		8 (7.6)	2 (6.1)	3 (8.8)	3 (7.9)	
1–2		35 (33.3)	16 (48.5)	9 (26.5)	10 (26.3)	
3–4		24 (22.9)	7 (21.2)	7 (20.6)	10 (26.3)	
5+		38 (36.2)	8 (24.2)	15 (44.1)	15 (39.5)	
Time with people centenarians did not live with	104					0.122^*^
None		16 (15.4)	8 (24.2)	4 (12.5)	4 (10.3)	
Once/week		18 (17.3)	9 (27.3)	6 (18.8)	3 (7.7)	
2–6/week		38 (36.5)	9 (27.3)	13 (40.6)	16 (41.0)	
One or more/day		32 (30.8)	7 (21.2)	9 (28.1)	16 (41.0)	
See or hear from relatives	98					0.317^*^
None		3 (3.1)	1 (3.0)	1 (3.1)	1 (3.0)	
1–2		22 (22.4)	7 (21.2)	11 (34.4)	4 (12.1)	
3+		73 (74.5)	25 (75.8)	20 (62.5)	28 (84.8)	
Have relatives as confidants	81					0.128^*^
None		21 (25.9)	9 (39.1)	6 (20.7)	6 (20.7)	
1-2		35 (43.2)	11 (47.8)	14 (48.3)	10 (34.5)	
3+		25 (30.9)	3 (13.0)	9 (31.0)	13 (44.8)	
See or hear from friends	83					0.134^*^
None		25 (30.1)	12 (50.0)	8 (26.7)	5 (17.2)	
1–2		18 (21.7)	4 (16.7)	7 (23.3)	7 (24.1)	
3+		40 (48.2)	8 (33.3)	15 (50.0)	17 (58.6)	
Have friends as confidants	71					0.015^***^
None		43 (60.6)	21 (87.5)	12 (42.9)	10 (52.6)	
1–2		19 (26.8)	2 (8.3)	11 (39.3)	6 (31.6)	
3+		9 (12.7)	1 (4.2)	5 (17.9)	3 (15.8)	
Feel alone	74					0.928^*^
Yes, many times		15 (20.3)	5 (20.8)	4 (15.4)	6 (25.0)	
Sometimes		22 (29.7)	8 (33.3)	9 (34.6)	5 (20.8)	
Almost never		18 (24.3)	5 (20.8)	7 (26.9)	6 (25.0)	
Never		19 (25.7)	6 (25.0)	6 (23.1)	7 (29.2)	
**Well-being**						
Satisfaction with life, mean (SD)	78	6.54 (1.93)	5.79 (2.06)	6.93 (2.00)	6.81 (1.59)	0.072^**^
Positive VOL (total), mean (SD)	81	16.7 (6.0)	11.1 (4.9)	20.0 (4.9)	18.2 (4.5)	<0.001^**^
Positive VOL factor 1, mean (SD)	81	9.39 (3.58)	6.08 (3.04)	11.1 (2.8)	10.5 (2.8)	<0.001^**^
Positive VOL factor 2, mean (SD)	81	7.32 (2.81)	5.07 (2.60)	8.84 (2.48)	7.71 (2.06)	<0.001^**^

* *Chi-square test*;

** *one-way ANOVA*;

*** *Fisher's exact test*.

## Discussion

Centenarians are an elite group, significantly exceeding the average life expectancy. This study explored the will to live and associated factors in a sample of these long-lived individuals by considering both quantitative indicators and qualitative data. The number of participants willing and unwilling to live longer was similar (31%) but lower than those without clear positioning (38%). Compared with the findings of studies of preferred life expectancy that also considered a non-response group, this was a very high percentage. For instance, in a study of 1,631 younger and middle-aged adults, Bowen and Skirbekk (2017) found that 15.9% of the sample did not clarify their preferred life expectancy. However, due to the lack of studies similar to the present one, whether the greater percentage was related to the centenarians' characteristics or the methodology of the study cannot be determined. Nevertheless, this group may have represented a stoic mindset in which individuals express a valuation of life *per se* and “as it comes” with discomfort or unwillingness to reflect about lifetime extension (Lang and Rupprecht, 2019).

Still, the qualitative exploration of this group's answers showed that almost everyone justified their lack of answer/positioning by mentioning God, stating that their remaining time to live was a matter that was not in “their hands” (i.e., one they could not control). This follows the idea that a sense of control through the sacred may come when life seems out of control (Wong et al., 2014). Although a sense of control is recognized as an important source of human life-strength, individuals who accept that declining control over environment comes with aging and focus on their ability to control their own internal states and behaviors demonstrate a more successful adjustment to aging (Hyer et al., 2011). Indeed, this group presented a satisfaction with life score very close to the group reporting willingness to live longer.

Regarding the reference to God, which was also present in the other two groups, religion and spirituality play an important role in the lives of older adults, as they help older people find meaning in later life (Frankl, 1963; Atchley, 2009; Wong et al., 2018) and are thus associated with how long one desires to live (Lang and Rupprecht, 2019). Different studies focusing on the centenarian population have confirmed the positive impact of religion and spirituality in well-being, which may be even more significant since this age group may fail to derive basic resources (Bishop, 2011). Archert et al. (2005) found that religiosity was one of the major themes that emerged from a qualitative analysis about adaptation and coping in the lives of centenarians; when asked about the most important thing in their lives, 58% of the respondents mentioned church and/or God. Interestingly, a female centenarian shared a sentence very similar to one of the participants in the present study, arguing that the future is held in God's hands (Archert et al., 2005). Furthermore, Manning et al. (2012) found that centenarians place considerable importance on divine support in their lives. This study found an interconnectedness of spirituality with religion for centenarians; in other words, these two constructs overlap. Through a phenomenological examination of life-satisfaction and compensatory strategies in Jewish-Canadian centenarians, Milevsky (2021) found that compensatory, cultural, and religious processes were imbued into several of the themes, such as “Maintaining connections with family, friends, and God” (p. 101) and “Remaining positive and kind” (p. 104). It is expected to have an intrinsic need to have hope that goes beyond this life and have faith either in something or in someone, which can be religiously oriented or without any religiosity (Saarelainen et al., 2020). But the huge presence of religion in centenarians' discourses found in the present study may echo the importance of church and military in shaping the lives of this older Portuguese generation (Birmingham, 2003), as well as the overall impact of religious beliefs, practices, and culture (Boerner et al., 2019).

The quantitative results of the present study revealed the significant contribution of health (pain frequency and SRH), social functioning (friends as confidants), and well-being (positive valuation of life). These findings confirmed that will to live is the summation of individuals' biopsychosociospiritual dimensions (Bornet et al., 2021) and depends on both external (e.g., social networks) and intra-personal factors (e.g., health and self-perceptions; Lawton et al., 1999). No sociodemographic variable was found to be relevant, which agreed with the findings of a scope review on will to live conducted by Bornet et al. (2021). Positive VOL had the strongest significant association with will to live, which was expected. Despite the great importance being attributed to variables like physical and health functionality in longevity and quality of life (Rowe and Kahn, 1997), some studies have emphasized the importance of psychological functioning and well-being, especially for very old individuals. For instance, a comparison of components in the World Health Organization's (WHO) active aging model by age group (< 75 years vs. ≥ 75 years) revealed the major relevance of the psychological component to the older age group (Paúl et al., 2017). Likewise, the operationalization of the successful aging model (Rowe and Kahn, 1997) in centenarians revealed the importance of subjective appraisals and psychological variables (Araújo et al., 2016).

The fact that the number of health conditions and levels of fatigue and functional capacity presented no significant association with will to live in this study supported the argument that individuals with problems related to physical health and functioning may be able to maintain subjective well-being. This agreed with the paradox of well-being, i.e., reporting experiences of positive psychological functioning despite decline in physical health, as the evidence of resilience in old age (Wiesmann and Hannich, 2014). Interestingly, pain and SRH, the two health factors with a significant impact on will to live, have also been associated with resilience in centenarians (Amaral et al., 2020). Gu and Feng (2018) argued that higher resilience could yield a greater protection for SRH and life satisfaction among centenarians compared with younger elderly groups. Thus, resilience may also be associated with will to live, as identified in younger groups (Bornet et al., 2021). This was supported by the qualitative analysis; aspects related to health, such as dependency, sense of burden, and fear of suffering, were referred to less than uselessness, annoyance, and loss of meaning by centenarians who were unwilling to live longer.

In advanced age, some aspects of purpose in life are more difficult to fulfill, such as having goals for the far future or feeling useful (Pinquart, 2002). This study found that these variables continue to be very important, specifically for (un)willingness to live. Conversely, will to live has strong, direct effects on well-being, including life and aging satisfaction (Jopp et al., 2017). The large influence of positive VOL on will to live meets Lawton et al.'s (1999) assumptions (i.e., years of desired life are mediated by VOL) in an age group in which this issue was not studied. Both existential beliefs and perceived control were higher in the group willing to live longer—that is, they represented important aspects for centenarians' reason for living, even under difficult conditions of functional impairment and disease. This confirmed that a “possible mechanism for the potency of VOL as a determinant of Years of Desired Life is the ability of people to adjust their standards for what is acceptable in everyday life in accord with changes in both their personal characteristics and the circumstances under which they live” (Lawton et al., 2001, p. 25).

Social factors also seem to have a contribution to will to live (Bornet et al., 2021), being an important source of meaning in life among older people (Saarelainen et al., 2020). Social relationships and support may be particularly important for centenarians (Boerner et al., 2016). The loss of friends and relatives that is typical in these long-lived individuals can make social contacts even more significant by reducing opportunities for (intra- and intergenerational) relationships (Randall et al., 2010). In the present study, the only social variable significantly associated with will to live was the number of friends as confidants. Thus, support—rather than size—may be the most significant aspect of social networks. Previous studies of the oldest old have shown the importance of having a close friend for independence (Pin et al., 2005) and well-being (Johnson and Barer, 1997). Indeed, maintaining a confidant is suggested as a strategy that centenarians use to compensate for losses and increase well-being (Araújo and Ribeiro, 2012). From the qualitative data, (social) disconnection and loneliness emerged as an important motive for losing will to live, as was also the case of feeling like a burden.

This last reason, which is typically referred to in studies on will to live (Bornet et al., 2021) since its related to the high burden of taking care of a person in the end of life, shows the need to acknowledge those who are supporting centenarians. The few studies that investigated will to live and end of life issues in centenarian's caregivers and offspring indicated concerns of family members that they'd become a burden for caregivers and would face the unavailability of family support if they became centenarians (Brandão et al., 2019). The fact that caregivers value this aspect so much, reinforces their potential burdens and needs. If being willing to live at 100 years old depends on the availability of social support, more must be invested in these caregiving offspring who are confronted with their own advanced age and the burdens of their parents' very old age (Eggert et al., 2020).

Despite the richness of this study's findings, some limitations should be considered. The cross-sectional design of the study prevented the ability to determine the direction of the relationships between variables, which could be of particular interest in this topic since willingness to live could be a predictor of well-being. Furthermore, those who shared their opinion (i.e., mostly individuals with mild or no cognitive impairment) represented only a part of the original sample, so these findings should not be generalized to the centenarian population.

## Conclusion

The current study added to the literature on will to live by presenting empirical research on an understudied population and focusing on a under-researched topic. A long life is an ambition and desire for many people, but the proportion of individuals who are willing to live longer at 100 years of age is the same as those who do not. Health factors appear to be significant in shaping such will, but social and psychological factors also play a role, which can be observed by the importance given to God and religious meaning and to connectivity. These results provide researchers suggestions for further investigation and highlight the importance of inquiring and understanding very old people's values and views on their will to live and their future wishes, and of creating conditions that promote very old people's meaning in life.

## Data Availability Statement

The datasets presented in this article are not readily available because this study is part of a larger. Requests to access the datasets should be directed to Laetitia Teixeira, laetitiateixeir@gmail.com.

## Ethics Statement

The study was approved by Portuguese National Data Protection Commission and the participants provided their written informed consent to participate in this study.

## Author Contributions

OR, LA, and LT were responsible for the study conception and design. OR supervised data collection and helped write the manuscript. LA wrote the manuscript. LA and OR performed the qualitative analysis. LT performed the quantitative data analysis and helped write the manuscript. RA critically revised the paper for important intellectual content. All authors contributed to the article and approved the submitted version.

## Funding

This article was supported by National Funds through Fundação para a Ciência e a Tecnologia (FCT) within the CINTESIS R&D Unit (reference UIDB/4255/2020).

## Conflict of Interest

The authors declare that the research was conducted in the absence of any commercial or financial relationships that could be construed as a potential conflict of interest.

## Publisher's Note

All claims expressed in this article are solely those of the authors and do not necessarily represent those of their affiliated organizations, or those of the publisher, the editors and the reviewers. Any product that may be evaluated in this article, or claim that may be made by its manufacturer, is not guaranteed or endorsed by the publisher.
